# An Intervention with Mineral Water Decreases Cardiometabolic Risk Biomarkers. A Crossover, Randomised, Controlled Trial with Two Mineral Waters in Moderately Hypercholesterolaemic Adults

**DOI:** 10.3390/nu8070400

**Published:** 2016-06-28

**Authors:** Laura Toxqui, M. Pilar Vaquero

**Affiliations:** Institute of Food Science, Technology and Nutrition (ICTAN-CSIC), Madrid 28040, Spain; laura.toxqui@ictan.csic.es

**Keywords:** sodium-bicarbonated mineral water, randomised controlled trial, cardiometabolic risk, cholesterol, fluid intake, human

## Abstract

Water intake is essential for health maintenance and disease prevention. The effects of an intervention with two mineral waters, sodium-bicarbonated mineral water (BW) or control mineral water low in mineral content (CW), on cardiometabolic risk biomarkers were studied. In a randomised-controlled crossover-trial, sixty-four moderately hypercholesterolaemic adults were randomly assigned to consume 1 L/day of either BW (sodium, 1 g/L; bicarbonate, 2 g/L) or CW with the main meals for eight weeks, separated by an eight-week washout period. Blood lipids, lipid oxidation, glucose, insulin, aldosterone, urine pH, urinary electrolytes, blood pressure, body weight, fluid intake, energy, and nutrients from total diet and beverages were determined. Total cholesterol, LDL cholesterol, and glucose decreased (*p* < 0.01), oxidised LDL tended to decrease (*p* = 0.073), and apolipoprotein B increased during the intervention, without water type effect. Energy and carbohydrates from beverages decreased since soft drinks and fruit juice consumptions decreased throughout the trial. BW increased urinary pH (*p* = 0.006) and reduced calcium/creatinine excretion (*p* = 0.011). Urinary potassium/creatinine decreased with both waters. Consumption of 1 L/day of mineral water with the main meals reduces cardiometabolic risk biomarkers, likely to be attributed to a replacement of soft drinks by water. In addition, BW does not affect blood pressure and exerts a moderate alkalizing effect in the body.

## 1. Introduction

Water intake is recognised to be essential for health maintenance and prevention of disease; however, the scientific evidence on this statement is very weak. There is no body storage for water and humans need to drink fluid several times a day. Water balance is achieved with wide individual intake variations depending on environmental temperature, physical activity and kidney regulation. In this regard, data of habitual water intake by healthy populations are limited and water requirements have been estimated in terms of adequate intake [1,2].

In addition to the amount of water, water composition should be also considered. For example, some mineral waters can significantly contribute to the daily intake of calcium or magnesium [3,4] and may play a role in the maintenance of bone mass and blood pressure. Our research group worked with a natural mineral water that is rich in mineral salts, and specifically contains bicarbonate, chloride, sodium, and free carbon dioxide. We observed that consumption of 1 L daily of this water for eight weeks as part of the usual diet reduced LDL cholesterol and cardiovascular risk indexes (total-cholesterol/LDL cholesterol and LDL cholesterol/HDL cholesterol) [5,6]. Moreover, soluble adhesion molecules (sICAM and sVCAM) and fasting glucose decreased in postmenopausal women, suggesting a possible role against cardiovascular diseases [5]. In two randomised, controlled, crossover trials, consumption of 500 mL of this water with a standard meal reduced postprandial lipaemia and insulin compared to a control water with low mineral content [7,8,9]. A reduction in cholecystokinin levels and lower gallbladder emptying in the first 2 h of digestion [9] was also found. Since this mineral water induces a slight metabolic alkaline effect, as demonstrated by changes in 24 h urine pH [6,10], an inhibitory effect in lipid absorption was suggested [9].

However, randomised, controlled trials to investigate the chronic cardiometabolic effects of waters are lacking. Thus, the aims of the present study were, (1) to examine the effects of an intervention with mineral water (1 L per day with the main meals for eight weeks) on blood lipids, lipid oxidation, glucose, insulin, aldosterone, and blood pressure; and (2) to examine if a sodium-bicarbonated mineral water, compared to a control mineral water (CW), has a beneficial influence on these parameters. The design is a crossover, randomised, controlled trial with two mineral waters differing in its electrolyte content, in moderately hypercholesterolaemic men and women.

## 2. Materials and Methods

### 2.1. Subjects and Design

Volunteers were recruited by different announcements in the press and university campus and through Web pages in Madrid, Spain. Men and women aged 18–45 years with total cholesterol > 5.2 and <7.8 mmol/L (>200 and <300 mg/dL) were recruited. Exclusion criteria were as follows: BMI < 20 and >30 kg/m^2^; being a usual consumer of carbonated mineral water; diabetes; hypertension; digestive, liver, or renal diseases; familial hypercholesterolaemia; being under medication that could affect lipid metabolism; and consumption of supplemented foods that could affect lipid metabolism (containing *n*-3 fatty acids or phytosterols).

A group of 280 men and women contacted the research group to receive information and 131 underwent the screening. Volunteers who did not meet the inclusion criteria or declined to participate were excluded (*n* = 59). Finally, a total of 72 subjects agreed to participate in the nutritional intervention and were randomly allocated in two groups using the random assignment method by a member of the research team who was not in contact with the volunteers. Participants were assigned to start consuming one water or the other following single randomisation using the RAND function of the Microsoft Excel software. All participants were instructed not to deviate from their regular habits and to maintain their normal diet, body weight, and exercise level.

The design was a randomised, crossover, controlled trial. A bicarbonated mineral water (BW) was compared with a control mineral water with low mineral content (CW). The composition of the waters is shown in Table 1. Both waters were provided in 0.5-L bottles without any label that could indicate their composition (Vichy Catalán, S.L., Barcelona, Spain). As BW contained 4.5 g of carbon dioxide per litre, and it was not possible to prepare a fizzy control water, the study was single-blinded.

Volunteers consumed 1 L/day of BW or CW water, as part of their usual diet with the two main meals, during an eight-week period, followed by an eight-week washout, and finally a second eight-week period of consumption of 1 L/day of the other water. All volunteers started the trial within a 10 days interval. The trial is registered as NCT02480816 [11].

This study was conducted according to the guidelines laid down in the Declaration of Helsinki and all procedures involving human subjects were approved by the Ethics Committee of the Spanish National Research Council and the Clinical Research Ethics Committee of Hospital Clínica Puerta de Hierro, Majadahonda, Madrid. Written informed consent was obtained from all subjects.

### 2.2. Compliance, Health, and Physical Activity Determinations

Study compliance was assessed monthly by questionnaires and a personal interview when volunteers underwent blood sampling. Questions included the amount or water consumed, moment of consumption, number of bottles consumed, and number of bottles left. As a compliance biomarker, urine pH at times 0 and 8 weeks of each water intervention period was used, according to previous experience of our research group (5, 6, 10). A questionnaire about smoking habits, health problems, medication use and changes in the normal routine was also filled out monthly. Physical Activity was measured by the Global Physical Activity Questionnaire (GPAQ).

### 2.3. Blood Pressure and Anthropometric Determinations

Blood pressure was measured monthly between 8:00 and 9:00 h in a quiet temperature-controlled laboratory using an automated digital oscillometric device (Omron model HEM 705-CP, Omron Corporation, Tokyo, Japan), and a mean of two readings was taken. Height was measured at the beginning of the study with a stadiometer incorporated into the scale. Waist circumference and body weight were measured monthly with a scale to a precision of 100 g (Seca, Hamburg, Germany). Body mass index (BMI) was calculated.

### 2.4. Dietary Assessment

Each subject’s dietary intake was assessed at baseline and at the end of each eight-week period. Volunteers completed a 72-h detailed dietary intake record specifying the types of food consumed, beverages, including water, and serving portions. Daily food, energy intake, nutrient intake, and energy provided by macronutrients were calculated using the Spanish Food Composition Database (DIAL software, Alce Ingeniería, Madrid, Spain). Beverages consumption, type, amount, and energy and macronutrients from beverages, was also recorded. These included tap water, mineral water, soft drinks, fruit juices, milk, black coffee or latte, tea, wine, beer, and distillate alcoholic beverages.

### 2.5. Sampling and Biochemical Analyses

Blood samples were collected at zero, four, and eight weeks of each period between 8:00 and 9:00 h and after 12 h fasting. Serum and plasma were obtained after centrifugation at 1000× *g* for 15 m and stored at −80 °C. Serum total cholesterol (T-chol), HDL cholesterol (HDL-chol), LDL cholesterol (LDL-chol), triglycerides, and glucose were determined by an automatic analyser (RA 2000; Technicon, Tarrytown, NY, USA). Insulin and apolipoproteins (Apo) A-I and B-100 were analysed by immunoturbidimetry (Behring Turbitimer, Barcelona, Spain) and aldosterone by ELISA (Biovendor, Brno, Czech Republic). The following ratios were calculated: T-chol/HDL-chol, LDL-chol/HDL-chol, HDL-chol/Apo AI, and LDL-chol/Apo B. Oxidised LDL was determined by ELISA (Mercodia, Upsala, Sweden). First morning urine samples were collected at zero and eight weeks, pH was measured and creatinine and the electrolytes sodium, potassium, and ionised calcium were analysed (9180 Electrolyte analyser, Roche, Switzerland). Urinary phosphate concentration was determined by a colorimetric assay (Abnova, Taipei, Taiwan). All determinations followed the ISO-9001-2000 quality control requirements.

### 2.6. Sample Size Calculation

Calculation of the sample size was based on the primary outcome of this study, LDL cholesterol, using values from our research group. In order to detect as significant a difference between treatments of 10%, with statistical power of 0.80 and a two-sided *p* = 0.05, a total of 65 subjects is needed (randomised to a 1:1 ratio to start with BW or CW). Results from complete dietary and biochemical data of 64 subjects (28 men and 36 women) are presented.

### 2.7. Statistical Methods

A normal distribution of variables was determined by the Kolmogorov-Smirnov test. Serum triglycerides values were log-normalised for statistical testing. Parametric data (biochemical, anthropometric, energy, and nutrient intake, and urine parameters are presented as mean ± standard deviation. Non-parametric variables are presented as mean, median, standard deviation, and interquartile range (IQR).

Linear general model effects was used to study the influence of treatment (basal 0, bicarbonated water 1, and control water 2), order (starting with BW 1 and starting with CW 2), and treatment × order interaction using time of analysis as within the subject measure. As the influence of order was not significant for any variable, data of the two periods of consumption of the same water were pooled and a two-way repeated measures analysis of variance (ANOVA) was carried out for the type of water (BW 1 and CW 2), time of analysis (zero, four, and eight weeks) and time × water interaction followed by post hoc Bonferrini test. The Wilcoxon signed-rank test was used to analyse the changes in beverages consumption.

Two-sided *p* values < 0.05 were considered significant. Data analyses were performed using SPSS version 22.0 for Windows (IBM, Armonk, NY, USA). The Bonferroni correction for multiple testing was used for the primary variable.

## 3. Results

**Figure 1:**
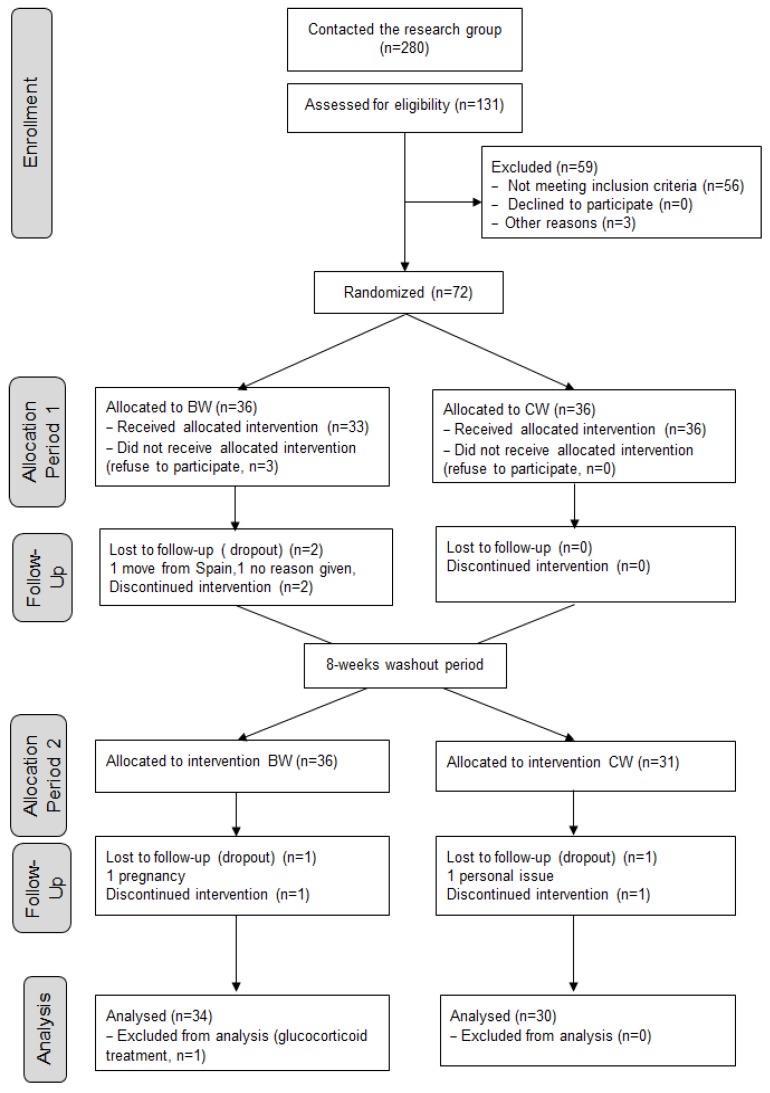
CONSORT flow diagram showing number of participants through each stage of the randomised crossover trial.

A total of 64 adults (56% women) aged 30.7 ± 7.5 years completed the study. Figure 1 shows the flow of participants through each stage of the trial according to the CONSORT diagram [12]. BMI values, 23.2 ± 2.8 at baseline and 23.0 ± 3.3 kg/m^2^ at week 8, did not show significant differences due to time or type of water. Mean waist circumference, 83.0 ± 10.9 and 83.4 ± 11.0 cm at baseline and week 8, respectively, did not vary, although men exhibited higher values than women (*p* < 0.001). Likewise, volunteers maintained lifestyle factors, such as physical activity and smoking habits, during the trial.

Table 2 shows cardiometabolic biomarkers at baseline and at four and eight weeks. T-chol, LDL-chol, glucose, and the cardiovascular risk indexes T-chol/HDL-chol, LDL-chol/HDL-chol, and LDL-chol/Apo B significantly decreased, while Apo B increased during time. Oxidised LDL tended to decrease (*p* = 0.073) and HDL-chol, triglycerides, Apo AI, the index HDL-chol/Apo AI, serum insulin, serum aldosterone, systolic and diastolic blood pressure did not vary throughout the trial. There was no time × water interaction for these variables, although aldosterone tended to decrease with BW, but not with CW (*p* = 0.08).

Total energy and macro- and micronutrient dietary intake is detailed in Table S1. Approximately 40% of the dietary energy was provided by lipids, with monounsaturated fat being the major contributor. No significant time × water effects were observed except for saturated fat, which showed an opposite trend in BW and CW, and for sodium, as BW was a source of this nutrient. At baseline, median total water intake (from food and beverages) was 2610 and 2049 mL/day and fluid intake (only from beverages) was 1898 and 1412 mL/day for men and women, respectively. Total water intake and fluid intake was higher at week 8 compared to baseline with both waters. Energy, macronutrient, alcohol, and fluid intake provided by beverages is presented in Table 3. A reduction in energy and carbohydrates from beverages throughout the intervention was observed, while lipids, protein, and alcohol remained unchanged. Total fluid intake increased during the trial with both mineral waters. Table 4 presents changes in beverages consumption at baseline and at week 8. Soft drinks and fruit juice significantly decreased, while total fluid intake significantly increased during the trial.

Urinary pH and calcium/creatinine ratio show significant time effect and time × water interactions (Table 5). BW consumption induced an increase in urinary pH (*p* = 0.006) and a decrease in calcium/creatinine ratio (*p* = 0.011), whereas CW did not change these parameters. Potassium/creatinine ratio decreased through time with both mineral waters and sodium/creatinine and phosphate/creatinine did not vary.

## 4. Discussion

Results show that chronic consumption of 1 L of mineral waters, BW or CW, with the main meals decreases T-chol, LDL-chol, and fasting glucose, and tends to decrease oxidised LDL, in moderately hypercholesterolaemic men and women. This could be partially attributed to a replacement of caloric beverages by water, since food consumption, physical activit, and anthropometric measures, such as BMI and waist circumference, did not vary throughout the trial.

Subjects were recruited according to their total cholesterol levels above 5.2 mmol/L (200 mg/dL) and could not be under treatment or present any metabolic or renal disease. Lifestyle factors including the habitual diet, beverages consumption, and biochemical changes induced by the intervention were monitored throughout the trial. As specific chronic, randomised, controlled trials with water are lacking, the present results are unique and novel.

LDL-chol, the main variable of this study, decreased by 5.8% and 3.2% with the consumption of BW and CW, respectively, and the differences between waters were not significant. Previous results of our research group showed that consumption of BW with a fat-rich standard meal compared to CW reduced postprandial lipaemia [8,9], which was attributed to a partial inhibition in lipid absorption [9]. In two chronic assays, significant decreases in LDL cholesterol and several markers of cardiovascular disease with BW were observed [5,6]. In those studies the design consisted in two consecutive two-month intervention periods, all volunteers consumed during the first period the CW and during the second period the BW and, therefore, each volunteer served as his or her own control. The present study design is of high quality, the subjects were randomised to begin with one or the other mineral water and after a washout period they were crossed to consume the other mineral water (see flow diagram in Figure 1), and it was obtained that the order in which the mineral waters were consumed was not a bias. All possible confounders were analysed, and in this regard the lack of effect of BW compared to CW on cardiometabolic markers could be partly attributed to the free-living diet, as intake of saturated fat was reduced in CW but not in BW (Table S1), and this could have counterbalanced the possible specific effect of BW.

The increase in Apo B levels and decrease in the LDL-chol/Apo B ratio with both waters observed in the present study suggests that the intervention increased the number of LDL particles and that these particles became smaller and more dense. However, this is not consistent with previous observations [6] and with the progressive decline in T-chol and LDL-chol. In this regard, it should be noticed that in addition to LDL particles, IDL and VLDL particles also contain Apo B and play a role in the reverse transport of cholesterol [13]. Otvos et al. [14], described four possible types of LDLs: large LDL particles with normal lipid content, small LDL with normal lipid content, and large and small LDLs with relatively cholesterol-deficient, triglyceride-rich cores. In our volunteers, although the exact lipid composition and lipoprotein size was not measured, it seems that LDL particles became smaller but with lower lipid content. Moreover, oxidised LDL tended to decrease (*p* = 0.07), triglycerides remained unchanged and the T-chol/HDL-chol and LDL-chol/HDL-chol ratios significantly decreased, thus a beneficial effect of the water intervention on lipoprotein metabolism is suggested. It should be also considered that these subjects had moderately high LDL-chol levels but their HDL-chol levels were within normal values, suggesting that the protective role of HDLs is unaffected.

A reduction of serum glucose during the intervention was also found. This could be explained by the replacement of other beverages by water as a result of the inclusion of the 500 mL portion per meal of the test mineral waters. Specifically, the consumption of soft drinks and fruit juices was decreased and, consequently, the energy and carbohydrates provided by beverages was also reduced. This result is outstanding considering that beverages contributed only to the 6.6% of total energy intake (see Table 3 and Table S1), much lower than in US adults and overweight Mexican women [15,16]. Inverse associations between sugar sweetened beverages consumption and energy intake have been reported in overweight women [17], and in children and youth [18]. This is in agreement with several studies that have associated the consumption of soft drinks and sugar-sweetened beverages with insulin resistance, obesity, and metabolic syndrome in different population groups [19,20]. Specifically, fructose in sugar sweetened beverages has been related to insulin resistance and adverse effects in lipoprotein metabolism [21]. We found that the intervention with the mineral waters induced a slight decrease in total energy intake from 2312 ± 611 to 2272 ± 539 kcal/day (*p* = 0.059, Table S1), therefore, negligible changes in food consumption could have also played a role.

The obtained fluid intake results confirm estimations by the European Food Safety Authority (EFSA) [22], concerning total water intake (from food and beverages) and fluid intake (only from beverage), but were lower than values suggested by The Institute of Medicine [23]. In this study, average total water intake was 2945 and 2276 mL/day and fluid intake was 2197 and 1581 mL/day for men and women, respectively. This means that approximately between 70% and 75% of total water intake was provided by beverages, being plain water the main contributor. Moreover, this trial using two different mineral waters induced an increase in total fluid intake. To date, the majority of data of water intake have been obtained from population surveys not specifically designed for assessing water intake. In this regard, our results are unique and add new information collected from a controlled water intervention in healthy adults.

Consumption of BW, which contains 2050 mg/L of bicarbonate, was capable to increase urinary pH, which confirms adherence to the water treatment, and reduced calcium excretion, suggesting a systemic alkalising effect that could help to counterbalance a possible dietary acid load [10,24,25]. It is known that Western diets (rich in protein and low in fruits and vegetables) promote a chronic, low-grade metabolic acidosis [26,27] and that the acidogenic potential of diets can be calculated by the net endogenous acid excretion (NEAP) score. NEAP scores of approximately 50 mEq/day for Western diets type [27], and 15 mEq/day for vegan diets [28], were reported. In the present trial, the calculated NEAP was 55.5 ± 16.4 mEq/day (mean ± SD), thus promoting alkalinisation by BW could be a favourable effect. Potassium is known to play important bone and cardiovascular protective effects. In the present study, although BW supplied more potassium than CW, it only contributed to approximately 1% of the total potassium intake which explains that similar effects were obtained with both waters. Concerning urinary calcium, BW may reduce its excretion by a direct renal mechanism, but it is also possible that intestinal calcium absorption decreased, as a result of lower solubility associated with higher pH during digestion and consequently urinary excretion decreased as a compensatory mechanism. Therefore, it is proposed that consumption of BW could help to compensate the acid/base imbalance and may protect from osteoporosis, diabetes, and metabolic syndrome, which are chronic diseases associated to acidogenic diets [24,29,30].

Finally, consistent with previous chronic studies [5,6], consumption of BW, which involves an extra intake of 1 g of sodium per day, did not affect blood pressure. This could be explained by the physiological response of aldosterone as fasting levels tended to be lower with BW at week 8 compared to baseline (Table 2). In two postprandial randomised, controlled trials, BW decreased aldosterone levels after 120 min of consumption [31,32] and increased sodium excretion in 7-h urine [31]. As changes in fasting urinary sodium by BW did not reach significant levels in the present study, we suggest that the inhibition of the renin-angiotensin-aldosterone system could be a short-term homeostatic mechanism which helps to maintain blood pressure in the long term.

Limitations of the study are that all volunteers were adults with moderately high cholesterol levels (T-chol above 5.2 mmol/L, 200 mg/dL), did not suffer from obesity, diabetes, or hypertension, but otherwise maintained healthy lifestyle habits. Therefore, the results cannot be extrapolated to conditions of cardiometabolic disease. In addition, genetic factors, lipoproteins composition, or particle size was not measured.

The main strength is the randomised, controlled, crossover design, which has the advantage of comparing changes in the same volunteer. The experimental and wash-out periods were eight weeks long and there were no carryover effects. Moreover, possible dietary and lifestyle confounders have been controlled and compliance was high. These results are novel and provide scientific data that can be used for establishing recommendations on fluid intake in the general population.

Research on water intake and metabolic effects of different types of water is an emerging research field. Further studies are needed to fully understand the long-term health implications and possible beneficial effects of water compared to other beverages, including soft drinks, and the effect of different types of mineral waters in populations with cardiovascular disease, obesity, metabolic syndrome, or other chronic disease.

## 5. Conclusions

Consumption of 1 L/day of both mineral waters, regardless of their composition, with the main meals by moderately hypercholesterolaemic adults, men and women, improves lipid profile and reduces serum glucose. This is attributed to a replacement of soft drinks.

Moreover, the sodium bicarbonate mineral water, which supplies 1 g of sodium and 2 g of bicarbonate per litter, does not affect blood pressure.

## Figures and Tables

**Table 1:** Composition of the mineral waters employed in the study.

	Control Water, mg/L (mmol/L)	Bicarbonated Water, mg/L (mmol/L) ^1^
Bicarbonate	74.9 (1.23)	2050 (33.55)
Chloride	4.8 (0.14)	622 (17.55)
Sulphate	10.6 (0.22)	50 (1.04)
Fluor	0.2 (0.01)	0.73 (0.04)
Calcium	22.6 (1.13)	20.8 (1.04)
Magnesium	2.8 (0.23)	5.8 (0.48)
Sodium	7.6 (0.33)	1090 (47.45)
Potassium	1.7 (0.04)	47.2 (1.21)

^1^ Contains 4.5 g of carbon dioxide per litter.

**Table 2:** Changes in cardiometabolic parameters of volunteers consuming bicarbonated water (BW) or control water (CW) for eight weeks.

Parameter	Baseline	4 Weeks	8 Weeks	Time	Time × Water
T-chol (mmol/L)					
BW	5.80 ± 0.77	5.70 ± 0.73	5.54 ± 0.73	0.004	0.24
CW	5.78 ± 0.71	5.66 ± 0.76	5.67 ± 0.73		
LDL-chol (mmol/L)					
BW	3.79 ± 0.81	3.70 ± 0.72	3.57 ± 0.70	0.001	0.18
CW	3.77 ± 0.71	3.62 ± 0.70	3.65 ± 0.74		
HDL-chol (mmol/L)					
BW	1.67 ± 0.47	1.68 ± 0.48	1.69 ± 0.50	0.52	0.52
CW	1.70 ± 0.54	1.66 ± 0.53	1.68 ± 0.53		
Triglyceride (mmol/L)					
BW	1.24 ± 0.66	1.21 ± 0.61	1.26 ± 0.65	0.26	0.13
CW	1.27 ± 0.50	1.22 ± 0.56	1.20 ± 063		
T-chol/HDL-chol					
BW	3.74 ± 1.15	3.63 ± 1.04	3.54 ± 1.04	0.049	0.24
CW	3.67 ± 1.05	3.69 ± 1.05	3.64 ± 1.06		
LDL-chol/HDL-chol					
BW	2.50 ± 0.97	2.40 ± 0.88	2.32 ± 0.85	0.036	0.21
CW	2.44 ± 0.89	2.42 ± 0.89	2.40 ± 0.90		
Oxidised LDL (mU/L)					
BW	56.7 ± 17.5	58.0 ± 18.6	54.7 ± 16.2	0.073	0.50
CW	59.2 ± 18.2	57.9 ± 16.5	56.8 ± 16.3		
Apo AI (mg/dL)					
BW	169.8 ± 30.8	169.1 ± 31.6	171.2 ± 32.5	0.52	0.69
CW	171.1 ± 31.8	169.3 ± 35.8	169.9 ± 32.5		
Apo B (mg/dL)					
BW	114.3 ± 20.7	119.5 ± 19.1	119.6 ± 18.9	<0.001	0.45
CW	114.5 ± 18.8	119.7 ± 21.1	122.2 ± 20.3		
LDL-chol/Apo B					
BW	1.28 ± 0.08	1.19 ± 0.10	1.15 ± 0.09	<0.001	0.36
CW	1.27 ± 0.08	1.17 ± 0.12	1.15 ± 0.09		
HDL-chol/Apo AI					
BW	0.38 ± 0.06	0.38 ± 0.06	0.38 ± 0.06	0.89	0.23
CW	0.38 ± 0.09	0.37 ± 0.06	0.38 ± 0.07		
Glucose (mmol/L)					
BW	4.95 ± 0.41	4.94 ± 0.46	4.80 ± 0.41	0.006	0.15
CW	4.90 ± 0.47	4.95 ± 0.47	4.88 ± 0.44		
Insulin (μIU/mL)					
BW	10.4 ± 5.0	10.3 ± 4.6	10.1 ± 4.5	0.18	0.16
CW	9.8 ± 4.4	11.4 ± 6.5	10.2 ± 5.8		
Serum aldosterone (pg/mL)					
BW	252.4 ± 83.0	-	241.3 ± 69.5	0.72	0.08
CW	251.7 ± 76.7	-	259.0 ± 85.2		
Systolic blood pressure (mmHg)					
BW	119.6 ± 12.7	119.0 ± 15.2	119.8 ± 14.0	0.41	0.45
CW	121.1 ± 14.5	119.2 ± 13.6	118.8 ± 13.7		
Diastolic blood pressure (mmHg)					
BW	72.6 ± 9.8	71.6 ± 8.9	72.6 ± 8.4	0.92	0.21
CW	71.4 ± 9.4	72.5 ± 9.8	72.0 ± 8.5		

Values are expressed as mean ± SD. As the influence of order was not significant by the linear general model for any variable, values of the two periods of the same water consumption were pooled. Time effect and time × water interaction by repeated measures ANOVA.

**Table 3:** Energy, macronutrients, alcohol, and fluid intake provided by beverages of volunteers consuming bicarbonated water (BW) or control water (CW) for 8 weeks.

	Baseline	8 Weeks	Time	Time × Water
Energy (kcal)				
BW	152 ± 157	118 ± 186	0.015	0.86
CW	163 ± 180	123 ± 129		
Protein (g)				
BW	1.1 ± 1.4	1.0 ± 1.5	0.12	0.40
CW	1.3 ± 1.5	1.0 ± 1.1		
Carbohydrate (g)				
BW	21.1 ± 22.0	14.7 ± 27.5	0.004	0.74
CW	20.3 ± 18.6	15.1 ± 14.9		
Lipid (g)				
BW	0.3 ± 0.4	0.4 ± 1.1	0.78	0.49
CW	0.3 ± 0.3	0.3 ± 0.4		
Alcohol (g)				
BW	8.2 ± 13.0	6.8 ± 12.1	0.11	0.68
CW	10.1 ± 18.0	7.7 ± 11.8		
Fluid (mL) *				
BW	1713 (975)	1849 (730) ^†^	0.020	-
CW	1579 (939)	1867 (609) ^†^	0.002	

Values are expressed as mean ± SD. As the influence of order was not significant by the linear general model for any variable, values of the two periods of the same water consumption were pooled. Time effect and time × water interaction by repeated measures ANOVA. * Values expressed as median (IQR). Difference between baseline and eight weeks by Wilcoxon signed-rank test; ^†^ Including 1 L of the test mineral waters.

**Table 4:** Changes in volunteers’ beverages consumption (mL/day) during the trial.

	Baseline			8 Weeks			*p*
	Mean	Median	IQR	Mean	Median	IQR	
Soft drinks	211.9	133.3	333	124.2	66.7	243	<0.001
Fruit juice	69.2	0	133	41.2	0	67	0.001
Milk	119.9	66.7	200	101.9	66.7	198	0.13
Black coffee	11.6	0	0	8.7	0	0	0.23
Latte, coffee with milk	128.5	116.7	233	130.2	116.7	200	0.77
Tea	24.9	0	0	17.3	0	0	0.54
Wine	25.6	0	0	18.4	0	0	0.14
Beer	126.3	0	133	91.3	0	110	0.056
Distilled alcoholic beverages	7.7	0	0	7.8	0	0	0.96
Total fluid intake ^1^	1850	1612	953	2012	1867	705	<0.001

^1^ Includes 1 L of the test waters (BW or CW) at week 8. IQR, interquartile range. Differences between baseline and week 8 by Wilcoxon signed-rank test.

**Table 5:** Changes in urinary pH and mineral excretion of volunteers consuming bicarbonated water (BW) or control water (CW).

Parameter	Baseline	8 Weeks	Time	Time × Water
Urinary pH				
BW	5.9 ± 0.6	6.2 ± 0.6	0.022	0.047
CW	5.9 ± 0.7	5.9 ± 0.5		
Calcium/creatinine				
BW	0.282 ± 0.152	0.233 ± 0.131	0.66	0.038
CW	0.287 ± 0.213	0.363 ± 0.450		
Potassium/creatinine				
BW	3.11 ± 1.78	2.86 ± 1.75	0.033	0.40
CW	3.15 ± 1.77	2.58 ± 1.22		
Sodium/creatinine				
BW	9.71 ± 5.12	10.82 ± 6.03	0.21	0.56
CW	8.82 ± 7.79	9.23 ± 5.42		
Phosphate/creatinine				
BW	2.98 ± 1.14	3.04 ± 1.04	0.15	0.39
CW	2.98 ± 1.03	3.25 ± 1.20		

Values are expressed as mean ± SD, ratios calculated in mmol/L. Time effect and time × water interaction by repeated measures ANOVA.

## Supplementary Materials

The following are available online at http://www.mdpi.com/2072-6643/8/7/400/s1, Table S1: Energy, macro- and micronutrient intake of volunteers BW or CW during 8 weeks.

## Abbreviations

The following abbreviations are used in this manuscript:

Apo	apolipoprotein
BW	sodium-bicarbonated mineral water
CW	control mineral water low in mineral content
