# Supplementary material for: An Intervention with Mineral Water Decreases Cardiometabolic Risk Biomarkers. A Crossover, Randomised, Controlled Trial with Two Mineral Waters in Moderately Hypercholesterolaemic Adults
Source: Nutrients. 2016 Jun 28;8(7):400. doi: 10.3390/nu8070400 (PMC4963876; doi:10.3390/nu8070400)
Supplement: Supplementary file 1 [file nutrients-08-00400-s001.docx]

**Supplementary Materials: An Intervention with Mineral Water Decreases Cardiometabolic Risk Biomarkers. A Crossover, Randomised, Controlled Trial with Two Mineral Waters in Moderately Hypercholesterolaemic Adults**

Laura Toxqui and M. Pilar Vaquero

**Table S1.** Energy, macro- and micronutrient intake of volunteers consuming bicarbonated water (BW) or control water (CW) for eight weeks.

| **Parameter** | **Baseline** | **8 Weeks** | **Time** | **Time × Water** |
| --- | --- | --- | --- | --- |
| Energy (kcal) | | | | |
| BW | 2312 ± 611 | 2272 ± 539 | 0.059 | 0.25 |
| CW | 2430 ± 623 | 2265 ± 629 |  |  |
| Protein (% energy) | | | | |
| BW | 16.2 ± 2.7 | 15.9 ± 2.9 | 0.53 | 0.59 |
| CW | 16.2 ± 2.7 | 16.2 ± 3.3 |  |  |
| Carbohydrate (% energy) | | | | |
| BW | 38.5 ± 6.9 | 38.5 ± 6.5 | 0.33 | 0.35 |
| CW | 38.5 ± 6.3 | 39.7 ± 5.7 |  |  |
| Lipid (% energy) | | | | |
| BW | 41.3 ± 6.7 | 41.8 ± 6.7 | 0.85 | 0.32 |
| CW | 40.9 ± 6.1 | 40.2 ± 6.6 |  |  |
| SFA (% energy) | | | | |
| BW | 13.0 ± 3.1 | 13.6 ± 3.3 | 0.48 | 0.005 |
| CW | 13.8 ± 2.7 | 12.8 ± 2.9 |  |  |
| MUFA (% energy) | | | | |
| BW | 18.4 ± 4.2 | 18.9 ± 4.3 | 0.92 | 0.18 |
| CW | 18.3 ± 3.7 | 17.1 ± 4.2 |  |  |
| PUFA (% energy) | | | | |
| BW | 5.4 ± 1.5 | 5.2 ± 1.2 | 0.60 | 0.099 |
| CW | 5.2 ± 1.4 | 5.6 ± 1.5 |  |  |
| Index (PUFA + MUFA)/SFA | | | | |
| BW | 1.9 ± 0.5 | 1.9 ± 0.5 | 0.54 | 0.085 |
| CW | 1.8 ± 0.4 | 1.9 ± 0.4 |  |  |
| Dietary fiber (g) | | | | |
| BW | 20.9 ± 10.0 | 20.9 ± 9.1 | 0.41 | 0.43 |
| CW | 21.6 ± 9.1 | 20.4 ± 10.7 |  |  |
| Total water intake (g) * | | | | |
| BW | 2363 (1124) | 2539 (908) | 0.012 | - |
| CW | 2312 (950) | 2523 (855) | 0.006 | - |
| Cholesterol (mg) | | | | |
| BW | 363.2 ± 132.6 | 384.8 ± 159.7 | 0.30 | 0.61 |
| CW | 348.1 ± 126.1 | 355.5 ± 161.0 |  |  |
| Calcium (mg) | | | | |
| BW | 900.8 ± 340.3 | 918.4 ± 377.8 | 0.40 | 0.16 |
| CW | 984.7 ± 369.0 | 915.0 ± 318.6 |  |  |
| Sodium (mg) | | | | |
| BW | 2714.2 ± 1060.9 | 3658.1 ± 744.9 | <0.001 | <0.001 |
| CW | 2792.8 ± 1074.5 | 2602.3 ± 964.3 |  |  |
| Potassium (mg) | | | | |
| BW | 3058.1 ± 1062.7 | 2966.8 ± 773.1 | 0.014 | 0.19 |
| CW | 3236.0 ± 805.5 | 2937.8 ± 1056.4 |  |  |
| Phosphate (mg) | | | | |
| BW | 1495.5 ± 417.0 | 1449.2 ± 408.9 | 0.009 | 0.16 |
| CW | 1632.3 ± 473.1 | 1477.9 ± 435.1 |  |  |
| Vitamin C (mg) | | | | |
| BW | 136.7 ± 95.5 | 122.6 ± 76.4 | 0.066 | 0.94 |
| CW | 138.5 ± 73.6 | 123.3 ± 84.7 |  |  |
| Vitamin D (µg) | | | | |
| BW | 3.8 ± 3.3 | 3.3 ± 2.6 | 0.25 | 0.86 |
| CW | 3.6 ± 3.2 | 3.2 ± 3.0 |  |  |
| Vitamin E (mg) | | | | |
| BW | 10.6 ± 4.4 | 10.3 ± 4.1 | 0.13 | 0.49 |
| CW | 10.9 ± 4.5 | 10.0 ± 4.3 |  |  |
| Retinol equivalent (µg) | | | | |
| BW | 979.0 ± 863.3 | 925.4 ± 484.4 | 0.26 | 0.85 |
| CW | 901.5 ± 391.7 | 826.0 ± 519.1 |  |  |

Values are expressed as mean ± SD. MFA, monounsaturated fatty acids; PUFA, polyunsaturated fatty acids; SFA, saturated fatty acids. * Includes water provided from solid foods and beverages; values are expressed as median (IQR), differences between baseline and 8 weeks by Wilcoxon signed-rank test.
